# A family history of serious complications due to BCG vaccination is a tool for the early diagnosis of severe primary immunodeficiency

**DOI:** 10.1186/1824-7288-39-54

**Published:** 2013-09-10

**Authors:** Pérsio Roxo-Junior, Jorgete Silva, Mauro Andrea, Larissa Oliveira, Fernando Ramalho, Thiago Bezerra, Altacílio A Nunes

**Affiliations:** 1 Department of Pediatrics, Ribeirão Preto Medical School, University of São Paulo, Avenida Bandeirantes, 3900, Ribeirão Preto, São Paulo 14049-900, Brazil; 2 Unit of Epidemiology, Ribeirão Preto Medical School, University of São Paulo, São Paulo, Brazil; 3 Department of Pathology, Ribeirão Preto Medical School, University of São Paulo, São Paulo, Brazil; 4 Laboratory of Medical Investigation Unit 56, Department of Dermatology, University of São Paulo, São Paulo, Brazil; 5 Department of Social Medicine, Ribeirão Preto Medical School, University of São Paulo, São Paulo, Brazil

**Keywords:** SCID, x-linked, BCG, Family members

## Abstract

Severe Combined Immunodeficiency (SCID) is one of the most severe forms of primary immunodeficiency (PID). Complications of BCG vaccination, especially disseminated infection and its most severe forms, are known to occur in immunodeficient patients, particularly in SCID. A carefully taken family history before BCG injection as well as delaying vaccination if PID is suspected could be a simple and effective method to avoid inappropriate vaccination of an immunodeficient child in some cases until the prospect of newborn screening for SCID has been fully developed. We describe a patient with a very early diagnosis of SCID, which was suspected on the basis of the previous death of two siblings younger than one year due to severe complications secondary to the BCG vaccine. We suggest that a family history of severe or fatal reactions to BCG should be included as a warning sign for an early diagnosis of SCID.

## Background

Severe combined immunodeficiency (SCID) represents a group of heterogeneous diseases that predominantly impair the development of T cells. The main manifestations are retarded growth and severe recurrent infections starting in the first year of life, caused by intra- and extracellular microorganisms. Unfortunately, most children are diagnosed only after the occurrence of severe infections and their complications, with a considerably worse prognosis. Severe pulmonary and hepatic infections are closely related to high morbidity and mortality. Thus, SCID is considered to be a pediatric emergency [1].

In Brazil, the BCG vaccine is given to all children during the neonatal period. Dissemination of *Mycobacterium bovis* (Mb) after BCG vaccination, with a fatal outcome, may occur in up to 30% of SCID patients [2].

We report a patient with a very early diagnosis of SCID, which was suspected on the basis of the previous death of two siblings younger than one year due to severe complications secondary to the BCG vaccine. We emphasize the importance of the family history for an early diagnosis of SCID.

This study was approved by the Ethics Committee of Clinics Hospital from the University.

## Case reports

The first pregnancy resulted in a boy born at term in good condition. The infant received the BCG vaccine at seven days of age and progressed without abnormalities until the third month, when he was hospitalized due to important weight loss, oral candidiasis and severe pneumonia, with no improvement with antibiotic therapy. Radiological examination demonstrated a bilateral infiltrate. The infant was transferred to the Intensive Care Unit due to important clinical worsening and died at five months of age. Autopsy revealed miliary tuberculosis (TB) with pulmonary, hepatic and splenic involvement. Laboratory tests: serial blood counts revealed anemia and lymphopenia (always below 1000 cells/mm^3^), and a blood culture revealed Mb growth.

Similarly, the second pregnancy resulted in a boy born at term and in good condition. The child received the BCG vaccine at seven days of age and developed right axillary adenomegaly and difficult healing of the vaccination wound. At two months of age, the child was treated on an ambulatory basis for acute viral bronchiolitis and pneumonia, with a poor clinical response. Respiratory discomfort and cough persisted. At four months of age there was worsening of breathing signs and symptoms and the onset of fever. Radiological examination revealed a bilateral infiltrate. The patient was admitted to the Intensive Care Unit due to respiratory insufficiency and died of septic shock. Autopsy revealed miliary TB with pulmonary, mesenteric, renal, hepatic and splenic involvement. Serial blood counts showed anemia and lymphopenia (always below 800 cells/mm^3^) and blood culture revealed Mb growth.

The parents (not consanguineous) were referred to a tertiary hospital for the investigation of possible untreated TB that might have caused the death of the children by vertical transmission. However, the investigation was negative for TB and immunodeficiency in both parents.

The third pregnancy resulted in a boy born at term in good condition. Because of the previous history, a blood count was performed at 12 hours of life, which demonstrated important lymphopenia (1000 cells/mm^3^). Consequently, the BCG vaccine was not applied and the child was referred to the Primary Immunodeficiency Outpatient Clinic for investigation. At seven days of life, laboratory tests were performed: a blood count revealed lymphopenia, quantitation of lymphocyte subtypes by flow cytometry revealed an important reduction of CD4^+^ and CD8^+^ cells (Table 1); a radiography and computed tomography revealed the absence of the thymus. The diagnosis of SCID T^-^B^+^NK^+^ was confirmed and transplantation with hematopoietic stem cells was indicated. At three months the patient showed a reduction of serum immunoglobulin levels and replacement therapy was started with intravenous gamma globulin. An allogeneic transplant of umbilical cord hematopoietic cells was performed at eight months. The patient coursed with no infections or graft versus host disease and with good weight-height development. The child is now four years old, has no infections and shows appropriate neuropsychomotor and weight-height development.

**Table 1:** Laboratory exams of the third patient performed at birth and at seven days of life

**Blood count**	**Birth**	**Seven days**
Hemoglobin g/mL (14–20)*	16.3	13.9
Hematocrit % (43–63)*	50	43
Platelets/mL (150.000–350.000)*	239.000	393.000
Leukocytes/mL (6400–11000)*	**24.400**	**6.200**
Neutrophils/mL (2000–18000)	**23180**	3410
Lymphocytes/mL (2700–5400)*	**1.000**	**1.600**
Eosinophils/mL (200–800)*	244	310
Monocytes/mL (700–1200)*	**0**	868
**Immunophenotyping**		
CD4^+^/mL (1700–2800)*	-	**0**
CD8^+^/mL (800–1200)*	-	**13**
CD19^+^/mL (500–1500)*	-	885
CD16/56^+^/mL (300–700)*	-	702

* Reference values according to age range.

Numbers in bold indicate outside normal ranges.

## Discussion

As we have shown in this article, a family history of severe complications in response to the BCG vaccine can be an important tool for the early diagnosis of SCID in infants. Many patients with immunodeficiency are asymptomatic at birth, so that an early diagnosis permitting the orientation of not using the BCG vaccine is very difficult. Not all SCID infants have problems with BCG. Some patients may only have localized infection, but dissemination is common and can occur without previous local signs [3].

Children with SCID are highly susceptible to early death due to systemic infections caused by attenuated vaccine strains, such as BCG [2].

The first two children had a similar outcome after BCG vaccination, progressing with delayed weight gain and severe respiratory infections. The detection of multisystemic miliary TB and the identification of Mb in blood cultures in both infants led to the consistent hypothesis that these severe manifestation were probably caused by the BCG vaccine. There are eventual reports of adverse reactions after immunization with BCG in previously healthy newborns, including atypical evolution of cicatrization, axillary adenopathy with or without suppuration, skin abscesses, and severe systemic manifestations. However, these conditions require rigorous clinical monitoring and immunological investigation, a fact that did not occur with these two infants. This fact underscores the importance of investigating the immunological competence of children younger than one year with a suspicion of severe infectious manifestations after receiving attenuated vaccines.

In the present investigation, because of the lack of confirmation of TB in the parents, the hypothesis of SCID was raised for the children, considering the early occurrence and severity of the infectious manifestations and their time relationship with the BCG vaccines, blood counts with persistent lymphopenia, and a family history of early death of a maternal brother due to infection [4]. In addition, the fact that all patients involved were males suggests an X-linked form of SCID such as the T^-^B^+^NK^+^ phenotype which was identified in our patient. Unfortunately, genetic diagnosis of SCID is still not available in Brazil, a fact that prevents us from reaching a more precise diagnosis. Most children with SCID are lymphopenic and neonatologists and pediatricians should be alert to this fact since healthy neonates and infants have a lymphocyte count exceeding 2500/mL. Thus, persistent lymphopenia below 2500/mL is a warning signal for SCID in this age group [5].

Referral of the parents to the University Hospital due to the fatal history of the previous children led to monitoring of the current pregnancy at a tertiary care hospital. A simple blood count at birth which detected profound lymphopenia was sufficient to raise the suspicion of SCID and to refer the patient to investigation. The diagnosis of SCID was made very early in the third child, a fact that permitted curative intervention by allogeneic hematopoietic cell transplantation with highly satisfactory results. The prognosis and survival of patients with SCID are directly related to the age at transplant. When the transplant is performed during the first three months of life and before the onset of infections, the survival is approximately 91% [2].

The most frequent clinical manifestation of BCG complication is disseminated infection in patients with SCID [6], as was observed with our patients. Therefore, the occurrence of disseminated TB due to the Mb strain of the vaccine should alert the pediatrician to the possibility of primary immunodeficiency (PID), especially SCID [3]. Mortality related to disseminated BCG infection alone or in association with other causes remains a serious problem for PID patients. Talbot et al. [7] reported a mortality rate of more than 70% for immunodeficient patients despite aggressive anti-TB therapy. Romanus et al. [8] suggested that vaccination should be postponed to 6 months of age in countries where there is a low general risk of neonatal TB. On the other hand, Brazil belongs to the group of 22 countries in the world that concentrate 82% of TB cases. The incidence of the disease in Brazil was 36/100,000 inhabitants in 2011, which has improved over the last decade but still remains very high. In this context, the BCG vaccine is still considered very important, especially against meningeal and miliary forms of TB, although controversy exists about its protective efficacy against the clinical form that has the greatest impact on TB control, namely pulmonary TB. Perhaps a carefully taken family history before BCG administration as well as delaying vaccination if a PID is suspected could be an effective method to avoid inappropriate vaccination of an immunodeficient child in some cases until the prospect of newborn screening for SCID has been fully developed.

The median age at diagnosis of SCID is between 4 and 7 months of life [9]. In many situations, the diagnosis in children without a suggestive family history is made later as a consequence of the multiple infections or of the severe reactions to the BCG vaccine [5]. In this respect, in order to facilitate an early diagnosis of PID in neonates, Griffith et al. [10] proposed the presence of at least one of these findings: a family history of infant death or immunodeficiency, delayed weight-height growth during the first months of life, infections with opportunistic pathogens without evidence of HIV infection, severe viral infections, absence of the thymus in a chest radiogram, microcephaly, and hospitalization due to septicemia associated with lymphopenia.

We conclude that, in those countries where BCG vaccine is used, pediatricians must be alert of its severe or fatal complications in other family members. This should be included as a warning sign and can be an important tool for the early diagnosis of SCID or of other severe forms of PID, contributing to a better prognosis.

## Consent

Written informed consent was obtained from the parents of the patient for publication of this Case report and any accompanying images. A copy of the written consent is available for review by the Editor-in-Chief of this journal.

## Competing interests

The authors declare that they have no competing interests.

## Authors’ contributions

PRJ conceived the study, and participated in its design and coordination and helped to draft the manuscript. JS participated in the design of the study. MA participated in the design of the study. LO conducted the case report. FR conducted the histological analyses. TB conducted the case report. AAN conducted the case report. All authors read and approved the final manuscript.
